# Inhibiting lung lining fluid glutathione metabolism with GGsTop as a novel treatment for asthma

**DOI:** 10.3389/fphar.2014.00179

**Published:** 2014-07-31

**Authors:** Marina Tuzova, Jyh-Chang Jean, Rebecca P. Hughey, Lou Ann S. Brown, William W. Cruikshank, Jun Hiratake, Martin Joyce-Brady

**Affiliations:** ^1^The Pulmonary Center, Boston University School of MedicineBoston, MA, USA; ^2^Department of Medicine, University of PittsburghPittsburgh, PA, USA; ^3^Department of Pediatrics, Emory University School of MedicineAtlanta, GA, USA; ^4^Institute for Chemical Research, Kyoto UniversityKyoto, Japan

**Keywords:** GGsTop, glutathione, lung lining fluid, asthma, IL-13

## Abstract

Asthma is characterized by airway inflammation. Inflammation is associated with oxidant stress. Airway epithelial cells are shielded from this stress by a thin layer of lung lining fluid (LLF) which contains an abundance of the antioxidant glutathione. LLF glutathione metabolism is regulated by γ-glutamyl transferase (GGT). Loss of LLF GGT activity in the mutant GGT^enu1^ mouse causes an increase in baseline LLF glutathione content which is magnified in an IL-13 model of allergic airway inflammation and protective against asthma. Normal mice are susceptible to asthma in this model but can be protected with acivicin, a GGT inhibitor. GGT is a target to treat asthma but acivicin toxicity limits clinical use. GGsTop is a novel GGT inhibitor. GGsTop inhibits LLF GGT activity only when delivered through the airway. In the IL-13 model, mice treated with IL-13 and GGsTop exhibit a lung inflammatory response similar to that of mice treated with IL-13 alone. But mice treated with IL-13 and GGsTop show attenuation of methacholine-stimulated airway hyper-reactivity, inhibition of Muc5ac and Muc5b gene induction, decreased airway epithelial cell mucous accumulation and a fourfold increase in LLF glutathione content compared to mice treated with IL-13 alone. Mice treated with GGsTop alone are no different from that of mice treated with saline alone, and show no signs of toxicity. GGsTop could represent a valuable pharmacological tool to inhibit LLF GGT activity in pulmonary disease models. The associated increase in LLF glutathione can protect lung airway epithelial cells against oxidant injury associated with inflammation in asthma.

## INTRODUCTION

The lung epithelial surface is bathed by a thin, continuous layer of fluid referred to as lung lining fluid (LLF). Its function has been described as a shield at the air–liquid interface to protect the lung epithelium from environmental stress (Cantin et al., 1987). The LLF provides an aqueous medium for the exchange of molecules within the surfactant system, a supportive milieu for the alveolar macrophage, a protective surface for the alveolar septum, and thin component of the blood-diffusion distance (Bastacky et al., 1995). Among the small molecule antioxidants present in LLF, glutathione (GSH) has received particular attention because its concentration exceeds that of the blood by 100-fold and its abundance exceeds that of LLF glutathione disulfide (GSSG; Cantin et al., 1987). In addition, the lung overall utilizes more glutathione than any other organ (Martensson et al., 1989). Cellular glutathione synthesis and extracellular export and metabolism impact overall glutathione homeostasis (Lieberman et al., 1996; Dalton et al., 2000; Shi et al., 2000). Metabolism of glutathione is regulated by gamma-glutamyl transferase (EC 2.3.2.2, GGT) and provides a cysteine source for cellular glutathione resynthesis (Hanigan and Ricketts, 1993). GGT is present in the lung as a soluble enzyme in association with surfactant phospholipids and controls turnover of the extracellular pool of glutathione in LLF (Joyce-Brady et al., 1994; Jean et al., 2002). The biological role of the LLF glutathione pool and its metabolism by GGT was revealed through our studies in a genetic mouse model of GGT deficiency, the GGT^enu1^ mutant mouse (Harding et al., 1997; Jean et al., 1999, 2002; Lowry et al., 2008). In a IL-13-driven model of experimental allergic asthma, GGT^enu1^ mutant mice were protected against asthma, despite the presence of cellular glutathione deficiency and oxidant stress in lung airway epithelial cells (Jean et al., 2002; Lowry et al., 2008). IL-13 is a well described pro-inflammatory cytokine that drives inflammation in a mouse model of experimental asthma (Grunig et al., 1998; Zhu et al., 1999). This cytokine-driven model evokes a similar degree of inflammatory cell infiltration into the lungs of normal mice and GGT^enu1^ mutant mice, thereby allowing study of their lung response to inflammation (Lowry et al., 2008; Joyce-Brady et al., 2012). We found that the GGT deficient GGT^enu1^ mouse was protected against asthma in this model by an increased size of the extracellular LLF glutathione pool which buffered oxidant species derived from inflammatory cells, and preserved airway epithelial cell barrier function. In turn, induction of mucin gene expression, mucous production and airway hyperreactivity were dramatically decreased in GGT^enu1^ mutant mice. Control mice did develop asthma but could be protected against this by inactivating their LLF GGT activity pharmacologically with acivicin, an irreversible GGT enzyme inhibitor. These data revived interest in the extracellular LLF glutathione pool and suggested that GGT activity in LLF is a novel target to treat asthma (Lowry et al., 2008; Joyce-Brady et al., 2009; Joyce-Brady and Hiratake, 2011).

Human pharmacotherapy with acivicin, however, is limited by neurotoxicity and cytotoxicity caused by an extended inhibitory activity against a number of other glutamine-dependent biosynthetic enzymes (Hidalgo et al., 1998; Joyce-Brady and Hiratake, 2011). These limitations led to the rational design of novel class of glutamate analogous γ-phosphono diesters as mechanism-based inhibitors of GGT. These compounds are based on stable analogs of the enzyme transition state and inhibit GGT activity with greater potency and specificity than acivicin (Han et al., 2006, 2007; Joyce-Brady and Hiratake, 2011). We focused on the lead compound from this class, now commercially available as GGsTop, for three reasons: lack of toxicity *in vivo*, physical stability *in vitro* and extended inhibitory activity against mammalian as well as bacterial GGT (Han et al., 2006, 2007). We hypothesized that GGsTop could attenuate asthma in the IL-13-driven model of allergic airway inflammation in mice and represent a valuable alternative to acivicin as a clinical therapeutic.

## MATERIALS AND METHODS

### INHIBITORY ACTIVITY OF GGsTop *IN VIVO*

A crystalline form of GGsTop was synthesized according to the previous report (Han et al., 2007). GGsTop is freely soluble in distilled water so a 10 mM stock solution was prepared, stored at -20^∘^C and aliquoted for experiments. The compound is now available commercially as GGsTop^TM^ from Wako Pure Chemical Industries, Ltd. (Osaka, Japan). In preliminary studies, GGsTop was assessed for its ability to inhibit GGT enzyme activity *in vivo* in mouse lung and serum at 0.5 mg/kg and 5 mg/kg. GGsTop was delivered via the peritoneum or through the trachea in a volume of 100 μL. GGT activity was assessed 2 h and 24 h afterward after delivery in bronchoalveolar lavage fluid and serum. Controls received phosphate buffered saline (PBS). GGT enzyme activity was assessed at room temperature using a standard method with γ-glutamyl-*p*-nitroanilide as substrate (Hughey and Curthoys, 1976; Joyce-Brady et al., 1994). Data are expressed as a percentage of the control group.

### MOUSE MODEL OF IL-13-DRIVEN EXPERIMENTAL ALLERGIC ASTHMA

C57BL/6 mice were housed in the Laboratory and Animal Science Center at Boston University School of Medicine under guidelines approved by the local Institutional Animal Care and Utilization Committee in protocol number AN-14284. Animals were fed Purina mouse chow and allowed access to water *ad libitum*. In each experiment, two groups of 6–8 mice were pre-dosed with saline or GGsTop dissolved in PBS via the trachea on day 0. Thereafter 3–4 mice were treated every 24 h for three successive days with either saline alone (control), saline with IL-13, saline with GGsTop, or saline with IL-13 and GGsTop. The IL-13 dose was five micrograms and the GGsTop was 0.5 mg/kg. On the final day, airway resistance (Rn) was measured using an intratracheal catheter on a Scireq flexivent apparatus (SCIREQ, Montreal, PQ, Canada) under appropriate anesthesia with pentobarbital. The tidal volume was 6–7 ml/kg, the positive-end expiratory pressure was 3 cm H_2_O, and the respiratory rate was 150 breaths/min. Basline airway resistance was measured after delivery of nebulized saline followed by increasing doses of nebulized methacholine Mch (5, 15, and 25 mg/ml). Data were normalized to the baseline level. Thereafter, lungs were either frozen to isolate RNA, inflation-fixed with paraformaldehyde for histology or lavaged to collect bronchoalveolar lavage fluid (Lowry et al., 2008).

### RNA ANALYSIS

Total RNA was isolated from frozen lung tissue, quantified by spectrophotometry and assayed for messenger RNA transcript abundance using standard real time PCR methodology as described (Lowry et al., 2008). The messenger RNA included two mucin genes, Muc5ac and Muc5b (Lowry et al., 2008) and the cytokine IL33 (Moffatt et al., 2010).

### HISTOLOGY AND CYTOLOGY

Standard techniques were used to fix lung tissue in 4% paraformaldehyde and to embed in paraffin. Sections were stained with periodic acid-Schiff (PAS) stain as described (Lowry et al., 2008). Cells in bronchoalveolar lavage were collected with low speed centrifugation, transferred to glass slides, stained with Giemsa, and visualized under light microscopy.

### BRONCHOALVEOLAR LAVAGE

Mouse lungs were inflated with 500 μL of PBS and centrifuged to sediment cells. The cell pellet was used to prepared cytospin preps and stained with Geimsa. The cell-free supernatant was combined with a sulfhydryl preserving solution and assayed for glutathione content by HPLC and normalized based on the urea dilution method as described previously (Jean et al., 2002; Lowry et al., 2008). Total glutathione content is reported.

### STATISTICS

Nominal data are presented as means with SE and analyzed by ANOVA and Dunnet’s or Bennett’s *post hoc* test as indicated. *P* values <0.05 were considered significant.

## RESULTS

### LUNG LINING FLUID GGT ACTIVITY IS INHIBITED BY GGsTop ONLY WITH INHALATION

**FIGURE 1:**
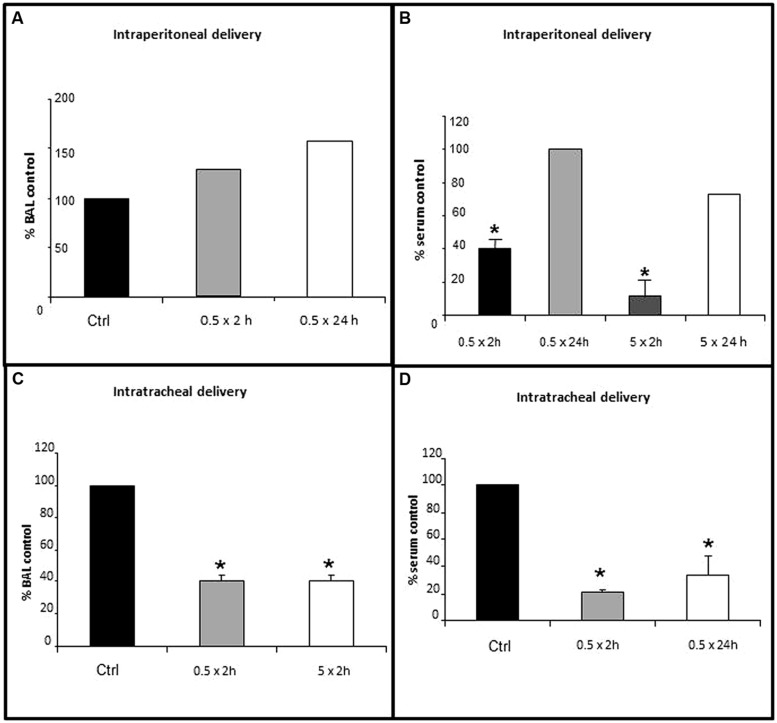
**Inhibition of lung lining fluid (LLF) GGT activity with delivery of GGsTop via the trachea, but not the peritoneum.** GGsTop was delivered thru the trachea and the peritoneum at doses of 0.5 mg/kg and 5 mg/kg. GGT activity was assessed in broncho-alveolar lavage fluid **(A,C)** and serum **(B,D)** as described in “Materials and Methods.” Data are expressed as percentage versus the control group. Significant differences at *p* < 0.05 are denoted by asterisks (*n* = 3). The data in 1A and those in 1B at 24 h are averages from *n* = 2 replicates.

Lung lining fluid GGT activity was not inhibited with GGsTop delivery thru the peritoneum at the 0.5 mg/kg dose (**Figure 1A**, *n* = 2) nor at the 5 mg/kg dose (same pattern, data not shown). This route did inhibit GGT activity in the blood (**Figure 1B**). The inhibition at 2 h was dose dependent with serum activity decreasing to 60% of control with 0.5 mg/kg (*p* < 0.05, *n* = 3) and 90% of control with 5 mg/kg (*p* < 0.05, *n* = 3). By 24 h there was a regain of serum GGT activity to 100% of control with the 0.5 km/kg dose (*n* = 2) and 67% of control with the 5 mg/kg dose (*n* = 2).

Two hours after GGsTop was delivered via the trachea, the 0.5 mg/kg dose and the 5 mg/kg dose each inhibited GGT enzyme activity in LLF to 40% of control (*p* < 0.05, *n* = 3, **Figure 1C**). Intratracheal delivery of GGsTop (0.5 mg/kg) also inhibited serum GGT activity to 20% of control at 2 h (*p* < 0.05, *n* = 3) and this decrease was still evident 24 h later (30% of control, *p* < 0.05, *n* = 3, **Figure 1D**). The 0.5 mg/kg dose was used for the IL-13 experiments and delivered through the trachea.

### GGsTop ATTENUATES IL-13 INDUCED AIRWAY HYPER-REACTIVITY

**FIGURE 2:**
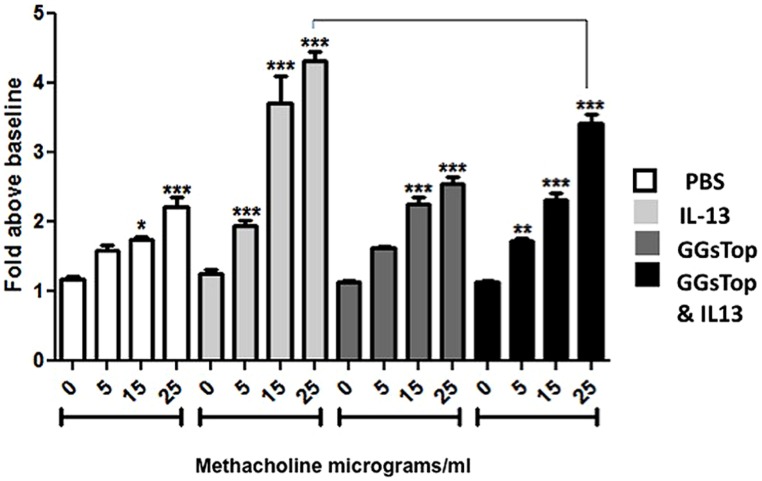
**GGsTop attenuates airway hyper-reactivity induced by IL-13.** Induction of airway hyperreactivity was measured by assaying airway resistance during graded methacholine challenge as described in “Materials and Methods.” Asterisks denote significant increase in airway resistance over baseline in each group (*p* < 0.05). IL-13 treatment increased airway resistance at each methacholine dose compared to saline control (*n* = 9/methacholine dose). Administration of GGsTop alone did not alter the airway resistance parameter versus the control group. Addition of GGsTop to IL-13, however, attenuated airway resistance at each dose of methacholine with a significant decrease at the highest dose (*n* = 9/methacholine dose, *p* < 0.0005).

Treatment of mouse lung with IL-13 caused a significant, and dose dependent increase in relative airway resistance following methacholine challenge compared to saline treated control mice (**Figure 2**). This pattern was attenuated at each methacholine dose when GGsTop was added to IL-13. The effect was significant at the highest dose (*p* < 0.0001, *n* = 9). Administration of GGsTop alone did not alter the airway resistance parameter versus the control group.

### GGsTop AUGMENTS TOTAL GLUTATHIONE IN LUNG LINING FLUID IN THE PRESENCE OF IL-13

**FIGURE 3:**
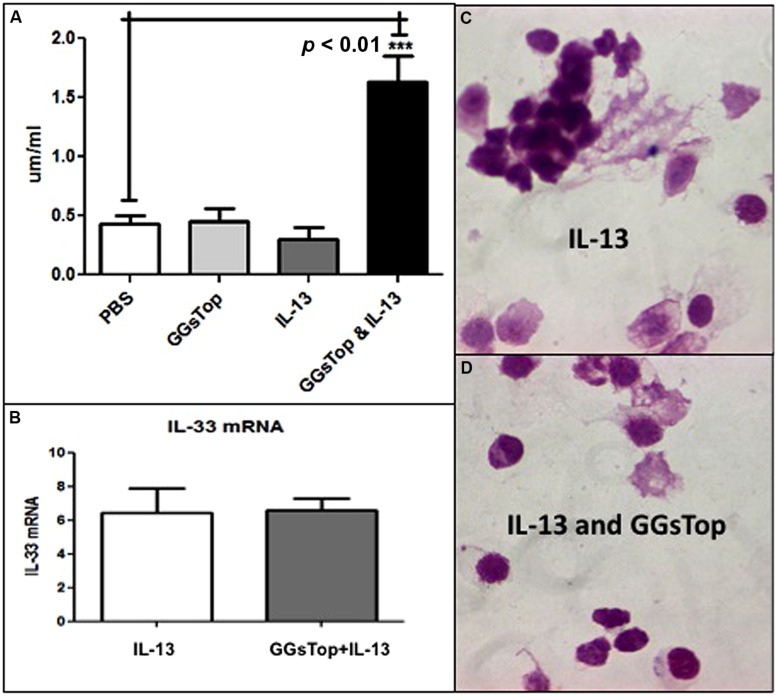
**(A)** GGsTop augments LLF glutathione in the presence of IL-13. Total Glutathione concentration (μM/ml) was measured in LLF at the end of the IL-13 experiment as described in “Materials and Methods” mice with the saline treated mice as control (0.4 μM/ml, *n* = 3). The glutathione concentration was similar to that of control after treatment with GGsTop (*n* = 3) and IL-13 (*n* = 3). It was significantly increased fourfold (1.6 μM/ml) in mice treated with GGsTop along with IL-13 (***p < 0.01, *n* = 3). **(B–D)** The IL-13 proinflammatory response is unaffected by GGsTop as IL-33 messenger RNA expression **(B)**, assessed by qRT-PCR in total RNA isolated from lung as described in “Materials and Methods”, is induced sixfold by IL-13 treatment alone (*n* = 3) and with GGsTop (*n* = 3), and an eosinophilic infiltrate predominates with IL-13 alone **(C)** and with GGsTop **(D)** as described by Lowry et al. (2008).

Total gutathione was measured in LLF at the end of the IL-13 experiment. In the absence of IL-13, GGsTop did not change LLF glutathione from that of the saline control (**Figure 3A)**. IL-13 treatment itself did not change LLF glutathione either. However, treatment with IL-13 and GGsTop increased LLF glutathione by almost fourfold (*p* < 0.05, *n* = 3).

### IL-13 DRIVEN INFLAMMATION IS UNAFFECTED BY GGsTop, BUT INDUCTION OF MUCOUS EXPRESSION IS ATTENUATED

Compared to control lung, exposure to IL-13 induced the level of lung mRNA transcript expression for IL-33 by sixfold (**Figure 3B**) and recruited eosinophils to the lung in the absence (**Figure 3C**) and presence (**Figure 3D**) of GGsTop.

**FIGURE 4:**
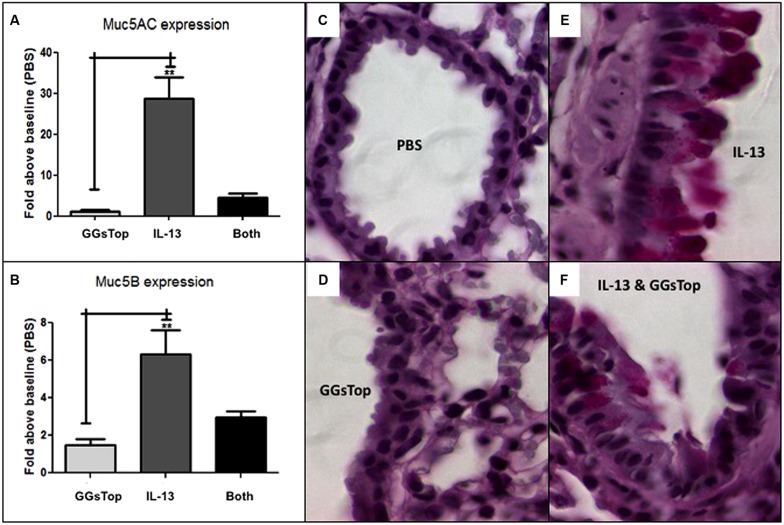
**GGsTop attenuates mucous production induced by IL-13.** Compared to saline control, lung level of Muc5ac **(A)** messenger RNA expression was not affected by GGsTop alone, but it was induced 28-fold by IL-13 treatment (**n = 3, *p* < 0.001). When GGsTop was delivered with IL-13, this level of messenger RNA induction was decreased sevenfold. **(B)** Similarly, Muc5b messenger RNA was induced sixfold **(B)** by IL-13 treatment (**n = 3, *p* < 0.01) and this was decreased twofold when GGsTop was delivered with IL-13. PAS stained lung **(C–F)** shows absence of mucous in control **(C)** and GGsTop treated **(D)** lung, and a diffuse mucous signal in airway epithelial cells after IL-13 treatment **(E)** but only rare mucous signal in a few cells when GGsTop was added to IL-13 **(F)** as described by Lowry et al. (2008).

IL-13 treatment induced the lung level of messenger RNA transcript expression for Muc5ac 28-fold (*p* < 0.05, *n* = 3, **Figure 4A**) and Muc5b sixfold (*p* < 0.05, *n* = 3, **Figure 4B**) versus control mice treated with saline alone. Compared to PBS treated lung (**Figure 4C**), PAS stain of IL-13 treated lung showed a diffuse signal for mucous in airway epithelial cells (**Figure 4E**; Lowry et al., 2008). When GGsTop was added to IL-13, induction of messenger RNA transcript for Muc5ac was decreased sevenfold (**Figure 4A**) and that of Muc5b twofold (**Figure 4B**) and PAS stained lung showed only rare airway epithelial cells with a mucous signal (**Figure 4F**). GGsTop treatment alone affected neither mucin gene expression nor mucous production (**Figures 4A,B,D**).

## DISCUSSION

Oxidant stress is believed to be important in asthma pathogenesis (Rahman et al., 2006). An inverse relationship has been noted previously in humans between the level of the antioxidant glutathione in LLF and airway hyperreactivity. That is a higher level of lung lining fluid glutathione is associated with lesser degree of airway hyperreactivity (Smith et al., 1993). The antioxidant glutathione is found in abundance in the lining fluid and protects the surface of the lung against oxidant stress (Cantin et al., 1987). LLF glutathione is metabolized by the enzyme GGT, which we showed is also present in lung lining fluid (Joyce-Brady et al., 1994). GGT expression in inducible LLF with inhalation of oxidant gas (Takahashi et al., 1997) and in many different cells in the lung during injury, including neutrophils, (Corti et al., 2012), bronchiolar Clara cells (Takahashi et al., 1997), and type 2 epithelial cells (van Klaveren et al., 1997). GGT-mediated glutathione metabolism provides cells with cysteine at the expense of the lung lining fluid glutathione pool. Interestingly, genetic deficiency of GGT in GGT^enu1^ mice induced an asthma-resistant phenotype in an IL-13-driven model of allergic airway hyperreactivity. Lung lining fluid glutathione content was augmented nearly 10-fold over that of normal mice at baseline, when GGT deficient GGT^enu1^ mice were exposed to this model of cytokine-driven inflammation. This enhanced antioxidant availability buffered inflammation-associated oxidant stress and attenuated airway epithelial cell EGF-receptor activation, induction of mucous hypersecretion and airway hyper-reactivity in GGT^enu1^ mice. Protection against asthma was conferred on normal mice by pharmacologic inhibition of LLF GGT with acivicin. Our data identified GGT as a target to treat asthma. We proposed this as a novel asthma treatment strategy to complement current asthma therapeutics which target inflammation (Lowry et al., 2008).

Our studies in the GGT^enu1^ mouse also demonstrated the value of this animal model in defining a biologic role for extracellular pools of glutathione in combating oxidant stress derived from inflammation. These observations can be translated into normal mice by augmenting their LLF glutathione thru inhibition of GGT enzyme activity (Joyce-Brady and Hiratake, 2011). In our original paper we successfully used acivicin to inhibit GGT activity. This inhibition is irreversible but certain adverse effects are already known to limit clinical acivicin use. It is non-specific and inhibits other glutamine amidotransferases as well as GGT (Joyce-Brady and Hiratake, 2011). It is also neurotoxic *in vivo* as shown in previous human clinical trials as a cancer therapeutic (Olver et al., 1998). GGsTop was actually rationally designed to eliminate both of these adverse drug interactions. Like acivicin, GGsTop is an irreversible inhibitor of GGT but is specifically designed for added potency and GGT selectivity (Han et al., 2006, 2007). Thus far GGsTop has exhibited no reported toxicity *in vivo* and has been utilized in a renal ischemia/reperfusion injury model in rats (Yamamoto et al., 2011). We did not observe any gross toxicity in normal mice with the doses used in this study.

Interestingly, systemic delivery of GGsTop at a single time inhibited serum GGT activity in a dose dependent manner and to a greater degree than airway delivery. But GGT activity reappeared within 24 h suggesting a more rapid clearance of GGsTop from the blood and replacement with active enzyme. GGT activity in LLF was totally unaffected by systemic delivery of GGsTop. Instead, GGsTop had to be delivered thru the airway to inhibit this pool of GGT activity within the lung. This was similar to our previous experience with acivicin (Lowry et al., 2008). The basis for this is not fully known. It is possible that first pass metabolism and clearance through the liver limits drug availability in the lung. Alternatively, both GGsTop and acivicin are hydrophilic and neither appears to penetrate the lung from the blood very efficiently. Several derivatives of GGsTop have been described and hydrophobicity may be related to ability to penetrate into the lung from the blood stream and inhibit LLG GGT activity (Han et al., 2006, 2007; Joyce-Brady and Hiratake, 2011). Nonetheless, airway delivery of GGsTop was effective in inhibiting GGT activity in LLF and in serum as well. Inhibition of serum GGT activity lasted up to 24 h after inhalation. Hence, there may be an advantage to inhaled GGsTop in that the lung can function as a GGsTop reservoir for the slow delivery of drug to the periphery to maintain inhibition of systemic GGT activity and augment plasma glutathione content and antioxidant defense which is known to be depleted during the pathophysiological state of sepsis (Malmezat et al., 2000).

Lung lining fluid GGT activity was inhibited equally well by the two doses (0.5 or 5 mg/kg) of GGsTop used in our study and we used the lower dose for all subsequent experiments. This result may reflect the fact that the lung has much less GGT activity overall compared to organs like the kidney or the pancreas even though GGT activity in the LLF is concentrated sevenfold over that of the lung as a whole (Joyce-Brady et al., 1994). Relative inhibition of GGT activity in LLF was somewhat less than that of the serum. The basis for this is not yet clear and further experiments will be required to determine if the level of GGT inhibition in the lung can be optimized further. Several derivates of GGsTop are available and can be tested for differential inhibitory activity against LLF GGT (Han et al., 2007; Joyce-Brady and Hiratake, 2011).

The advantage of this IL-13 cytokine driven model of allergic airway inflammation is in the similar levels of inflammatory cells that are recruited to the lung. This permits one to focus on lung airway epithelial cells and their response to the inflammatory process (Joyce-Brady et al., 2012). Similarity of the inflammatory response after IL-13 delivery to the lung in this study is suggested by the comparable levels of induction of IL33 cytokine messenger RNA and eosinophilic infiltrate in bronchoalveolar lavage fluid compared to untreated controls in the absence or presence of GGsTop. However, that the lung responded differently after IL-13 delivery in the presence of GGsTop was shown in three ways. Methacholine-stimulated airway hyperreactivity was significantly attenuated in mice treated with IL-13 and GGsTop, whereas it was induced in mice treated with IL-13 alone. Induction of mucin gene expression, assayed as messenger RNA expression for Muc5ac and Muc5b, and mucous accumulation, visualized by PAS staining, were significantly attenuated in the lungs of mice treated with IL-13 and GGsTop, but all were induced dramatically in mice treated with IL-13 alone compared to control mice. In all of our experiments using this IL-13 model, we hypothesize that effective buffering of inflammation-derived reactive oxygen species by the augmented glutathione pool in lung lining fluid protected airway epithelial cells from injury. LLF total glutathione was augmented in the IL-13 treated GGT^enu1^ mouse and in the normal mouse treated IL-13 and GGsTop, although to a lesser degree. We believe this protection preserved airway epithelial cell barrier function, prevented mucin gene induction and attenuated development of airway hyperreactivity (Lowry et al., 2008).

Other investigators have also proposed inhibiting GGT activity as a treatment for human diseases besides asthma including cancer (Hanigan, 1998), cardiovascular disease (Emdin et al., 2005), and *cis*-platinum-induced nephrotoxicity (Hanigan et al., 2001). However, augmentation of glutathione availability was not the proposed mechanism of action. Rather, it was decreased production of a pro-oxidant glutathione metabolite Cys-Gly (Emdin et al., 2005) or a toxic GGT-dependent *cis*-platinum-glutathione conjugate (Townsend and Hanigan, 2002; Townsend et al., 2003). Hanigan has even developed a novel alternative, non-toxic, and non-competitive but reversible GGT inhibitor that could also be used clinically to replace the toxicity of acivicin (King et al., 2009). Nonetheless, GGsTop, a mechanism-based and irreversible inhibitor of GGT, has been shown to be a non-toxic alternative to acivicin in a recent study where it was used to inhibit GGT activity and attenuate injury in a rat kidney ischemia/reperfusion model (Yamamoto et al., 2011). Together these studies all support the use of novel GGT inhibitors as potential therapeutics in GGT-dependent diseases. In addition, our findings may be relevant for other lung diseases that involve inflammation and perturb the extracellular LLF glutathione pool such as cystic fibrosis, adult respiratory distress syndrome, and chronic obstructive pulmonary disease (Cantin and Begin, 1991; Hull et al., 1997). Our strategy to inhibit lining fluid GGT activity is centered on the augmentation of antioxidant defense by increasing glutathione availability in the lung lining fluid, thereby preserving the barrier role of the lung epithelium and normal lung function. GGsTop and its derivatives provide a new avenue for study toward that goal.

## AUTHOR CONTRIBUTIONS

Provided GGsTop and handling guidelines (Jun Hiratake); performed GGT activity analyses and interpretation (Rebecca P. Hughey, Martin Joyce-Brady); analyzed lung lining fluid glutathione (Lou Ann S. Brown, Martin Joyce-Brady); RT-PCR analyses for gene expression (Jyh-Chang Jean); analyzed methacholine-stimulated hyper-reactivity with SCIREQ apparatus, prepared and analyzed bronchoalveolar lavage cytospins and lung histochemistry (Marina Tuzova, William W. Cruikshank, Martin Joyce-Brady); reviewed manuscript (Marina Tuzova, Jyh-Chang Jean, Rebecca P. Hughey, Lou Ann S. Brown, William W. Cruikshank, Jun Hiratake); wrote manuscript (Marina Tuzova, Martin Joyce-Brady).

## Conflict of Interest Statement

The authors declare that the research was conducted in the absence of any commercial or financial relationships that could be construed as a potential conflict of interest.
